# Proteomic Changes in Antioxidant System in Strawberry During Ripening

**DOI:** 10.3389/fpls.2020.594156

**Published:** 2020-12-23

**Authors:** Jun Song, Leslie CampbellPalmer, Mindy Vinqvist-Tymchuk, Sherry Fillmore, Charles Forney, Honghui Luo, Zhaoqi Zhang

**Affiliations:** ^1^Agriculture and Agri-Food Canada, Kentville Research and Development Centre, Kentville, NS, Canada; ^2^College of Horticulture, South China Agriculture University, Guangzhou, China

**Keywords:** *Fragaria* × *ananassa*, fruit ripening, quality, multiple reaction monitoring, OFFGEL, proteomics

## Abstract

To investigate the strawberry antioxidant defense system during fruit ripening, a targeted quantitative proteomic approach using multiple reaction monitoring (MRM) was developed to investigate targeted proteins in the antioxidant enzyme system in strawberry fruit. We investigated 46 proteins and isoforms with 73 identified peptides which may be involved in this antioxidant enzyme system. Among the proteins that changed during ripening, aldo/keto reductase (AKR), superoxide dismutase (SOD) and glutathione transferase (GT) increased significantly, while dehydroascorbate reductase, 2-Cys peroxiredoxin, catalase (CAT), 1-Cys peroxiredoxin and L-ascorbate peroxidase (APX) decreased significantly. These results suggest that fruit ripening of strawberry activates the enzymes of an SOD/glutathione metabolism system. The methodologies used in this study will be useful for systematically characterizing the role of antioxidant enzymes in fruit ripening of other plants.

## Significance

Gaining insights into the fundamental mechanisms at molecular level in association with the antioxidant enzyme system and its regulation during strawberry fruit ripening is challenging caused by limited research tools and lack of established hypotheses. Despite intensive molecular and biochemical research into fruit ripening, quantitative proteomic studies through an MRM approach have been rare. Using LC-MS/MS analysis and developing quantitative MRM, this study provides a systematic and multi-targeted investigation of the antioxidant enzymes in strawberry fruit at different ripening stages. Targeted detection of proteins in association with the antioxidant enzyme system used for quantitative approaches of MRM was developed. It is possible to examine their changes as the strawberry fruit matures. Our results demonstrate the usefulness of this technique for the analysis of regulatory proteins. These results provide evidence of the dynamic changes in SOD/glutathione metabolism that occur during fruit ripening.

## Introduction

Strawberry (*Fragaria* × *ananassa*) fruit is one of the popular fruit in the world, with significant economic benefits to the fruit and food industry. It is estimated that the world production of strawberry fruit was 9.1 million tons, with an associated value of $2.9 billion in 2016 (FAOSTAT, 2016). The attractiveness of strawberry fruit to consumers is dependent on a number of quality attributes including color, flavor and nutritional value. From a post-harvest perspective, however, strawberries have a relatively short market life. Research focusing on fruit ripening and quality is therefore important in order to develop new storage and marketing strategies to maintain and improve the quality and shelf-life of strawberry fruit.

Significant research has investigated the biology of fruit ripening and senescence in strawberry fruit resulting in the identification of a number of genes involved in ripening such as anthocyanin biosynthesis, cell wall degradation, sucrose and lipid metabolism, protein synthesis and degradation, and respiration (Manning, 1998). In addition, a number of genes were identified that encode proteins believed to participate in the signal and regulation cascades involved in maturation of achene maturation as well as acquisition of stress. As well, genes associated with stress, cell wall metabolism, DNA/RNA/protein interaction and primary metabolism were highly represented in the receptacle tissues (Nam et al., 1999). Various studies have investigated strawberry fruit during ripening and identified regulating genes involved in ethylene reception (Trainotti et al., 2005), firmness (Redondo-Nevado et al., 2001; Villarreal et al., 2008), allergens (Hjernø et al., 2006) and anthocyanin biosynthesis (Griesser et al., 2008; Carbone et al., 2009; Kadomura-Ishikawa et al., 2015). Metabolomic studies empolying gas chromatography-mass spectrometry (GC/MS) and liquid chromatography-mass spectrometry (LC/MS) revealed dynamic changes of primary and secondary metabolies reflecting organ and development stages of strawberry fruit separately, in achene and receptacle during ripening of strawberry fruit (Fait et al., 2008). Strawberry is recognized as a non-climacteric fruit, which ripens without the autocatalytic ethylene response (Symons et al., 2012). It was reported that the fruit was able to continue ripening after being detached at the green stage, however, reduced content of malic acid, glucose, fructose and sucrose content was found to be lower than fruit ripened on the plant. This would indicate that normal fruit ripening requires a continuous supply of photosynthetic assimilates, water and nutrients by the plant (Van de Poel et al., 2014). It has been shown that auxins and abscisic acid (ABA) related metabolism and signaling in strawberry play an important role in fruit ripening and strawberry has been used as a model of non-climacteric fruit ripening (Medina-Puche et al., 2016). Hormone interplay was reported during strawberry fruit ripening and an increase in carbohydrates, phenylpropanoids, flavonoids, glutathione, and methionine is controlled by ABA, while auxins are mainly responsible for the receptacle development. Ethylene and gibberellins seem to play a minor role in strawberry ripening (Medina-Puche et al., 2016). Gene expression studies on strawberry fruit using auxin treatment showed the reduced genes expression in flavonoids metabolism, pigmentation as well as tubulin and profilin (Aharoni et al., 2002). In addition, it was also reported that the strawberry fruit ripening is induced by a transcriptional program in response to oxidative stress. In non-climacteric fruit, it was proposed that gene expression is induced to cope with oxidative stress that occurs during ripening. Therefore one of the strawberry ripening transcriptional programs is an oxidative stress-induced process (Aharoni et al., 2002). From both fruit ripening physiology and nutritional perspectives, research into redox and antioxidant enzymes in association with strawberry fruit ripening is important (Decros et al., 2019).

Although numerous biochemical and physiological studies have been conducted to reveal the mechanism of fruit ripening and senescence of strawberry, limited proteomic data is available to provide in-depth information in our understanding of strawberry fruit ripening and senescence beyond the above identified genes (Bianco et al., 2009; Palma et al., 2011; Li et al., 2013).

Proteomics is the study of “the entire protein complement expressed by a genome in a cell or tissue” (Pandey and Mann, 2004). Proteomics has become an essential tool of “omic” approaches and is contributing to a better understanding of biological systems by measuring gene products, which are the active agents in cells. More importantly, modifications of proteins and post-translational modifications can be determined only by proteomic methodologies (Fridman and Pichersky, 2005). Proteomic studies using LC/MS, have been developed and applied to investigate the fruit ripening, physiological disorder and abundance of allergens at proteomic level. These studies investigated and identified proteins in association with fruit ripening, cell wall metabolism, natural defense and allergens in apple and strawberry fruit (Pedreschi et al., 2007; Bianco et al., 2009; Zheng et al., 2013). Employing labeling techniques, a quantitative proteomic study was conducted and identified a number of significantly changed proteins in association with fruit ripening. Significantly increased proteins associated with various metabolic pathways including flavonoid/anthocyanin biosynthesis, volatile biosynthesis, antioxidant metabolism, stress responses and allergen formation were found. Meanwhile, proteins that are involved in methionine metabolism, antioxidant-redox, energy metabolism and protein synthesis decreased during fruit ripening. Quantitative proteomic research identified certain proteins that increased during fruit ripening, including aldo/keto reductase (AKR), quinone oxidoreductase and ascorbate peroxidase (APX). Another group of proteins found to decrease as ripeness advanced included 1-Cys peroxiredoxin (PRX), 2-Cys PRX, isoflavone reductase related protein, as well as cytoplasmic SOD[Cu/Zn] (Li et al., 2013). Selected reaction monitoring (SRM) and/or multiple reaction monitoring (MRM) technologies have come out as a major development for targeted quantitative proteomic analysis (Lange et al., 2008). In an SRM experiment, it is usually conducted on a triple quadrupole instrument equipped with two mass filters. Therefore, it can detect and monitor predefined precursor ion and one of its fragments. As a result, series of transitions (precursor/fragment ion pairs) can be established and analyzed. With the help of LC and the retention time of peptides, targeted peptides can be quantitatively measured in a complex biological sample (Song et al., 2015a). MRM studies offer very good sensitivity and a wide dynamic range, which provide new technological advantages in plant biotechnological and biological applications to quantify targeted proteins. Targeted quantitative proteomic studies using MRM reported the key regulating points responsible for the biosynthesis of anthocyanins, flavonoids and volatile biosynthesis pathways in strawberry fruit in correlation with fruit ripening (Song et al., 2015a, b). These results demonstrate that strawberry fruit ripening is regulated by a complex network involving multiple active processes. With development of analytical techniques, specialized pathways including redox and antioxidant enzymes in fruit such as apple have been reported (Qin et al., 2009). All these findings provided some initial understanding of the involvement of redox systems in the physiological responses of fruit, including regulation of fruit ripening and senescence.

In order to analyze the changes of antioxidant enzyme isoforms involved in redox-antioxidant systems during strawberry fruit ripening, we reanalyzed results previously collected from OFFGEL electrophoresis (OGE) fractionation of a complex mixture of strawberry fruit peptides analyzed with LC-MS/MS (Yang et al., 2014). We evaluated and established the MRM transitions for those peptides, and then quantified proteins in two strawberry cultivars at three different ripening stages. The objectives of this study were to quantitatively determine the changes of protein abundance in association with redox and antioxidant system at different stages of fruit ripening, and to demonstrate links between redox protein changes and ripening physiology in order to gain better insight of strawberry fruit ripening at the proteomic level.

## Materials and Methods

### Fruit Materials

Strawberry (*Fragaria* × *ananassa*, Cv. “Honeoye” and “Mira”) fruit were planted at the Kentville Research and Development Centre, Agriculture and Agri-Food Canada, Kentville, NS, Canada. Fruit were harvested at three harvest maturities (white, pink, and red). Collection of fruit samples has been previously described (Li et al., 2013). Briefly, fruit maturities were determined by using 14, 18, and 22 days after anthesis, respectively. The experiment was conducted in triplicates with three harvests. For each cultivar and harvest, three blocks of plants were randomly selected, harvested and pooled. Samples were frozen in liquid nitrogen, then ground to a fine powder in using a stainless steel blender followed by a mortar and pestle. Ground tissue were stored at −85°C until used.

### Protein Extraction

Protein sample preparation and extraction were previously reported employing a phenol protocol (Zheng et al., 2007). Quantification of protein, tryptic in-solution digestion and desalting were described previously (Zheng et al., 2007).

### Protein Digest Fractionation

Protein digest fractionation was performed on a 3100 OFFGEL fractionator equipped with 24-wells (Agilent Technologies, Palo Alto, CA, United States). The detailed procedure and settings were similar to the manufacturer’s protocol with minor modifications and were previously reported (Yang et al., 2014).

### LC/MS/MS and Data Analysis

Liquid chromatography-mass spectrometry analysis and parameter have been previously described (28 and 29). Briefly, chromatographic separation of peptides was conducted on a nano-flow LC system (Ultimate 3000, Dionex, Sunnyvale, CA, United States). MS analysis was analyzed on a QTRAP 4000 (AB Sciex, Toronto, ON, Canada) on all 24 fractions via OFFGEL. Collected MS/MS data was manually inspected and searched against NCBI *viridiplantae* entries with 903,371 sequences (NIH, Bethesda, MD, United States) and Swiss-Prot database with 515,203 sequences (Sprot version 57.15) on Mascot (Version 2.4, Matrix Science, London, United Kingdom). Peptide ion scores greater than 43 were accepted as identification as with significant hits or extensive homology (*p* < 0.05).

### MRM Assays of Selected Proteotypic Peptides of Strawberry Fruit

Similar approaches were taken to select MRM transitions for proteins in relation to redox and antioxidant system as previously reported for volatile and anthocyanin biosynthesis (25 and 26). Briefly, selections of protein sequences for all proteins and isoforms in association with redox and antioxidant systems were performed. Further evaluation of MRM transitions were conducted on the peptides with at least 7 amino acids and without methionine and cysteine. To confirm the identification of proteins, Peptides were blasted against the NCBI database and Uniprot^1^. The selected peptides and proteins for MRM study are provided in Supplementary Table S1.

Validation of the MRM assay was conducted on a mass spectrometer (QTRAP 4000, AB Sciex, Toronto, ON, Canada) with MRM mode. When an MRM trace was detected, a full MS2 spectrum was triggered to an acquisition using a threshold of 100 ion counts, which was operated with Q1 and Q3 at unit resolution (0.7 m/z half maximum peak width). Detailed MRM settings and parameters were reported previously (24, 25, and 26). Protein Pilot v1.5.2 and MRMPilot v2.0 (AB Sciex, Toronto, ON, Canada) and Skyline (v2.5)^2^ were used to characterize and validate the MRM transitions (25 and 26).

Two synthesized peptide standards, which were isotopically labeled by incorporating ^13^C/^15^N on Leu were purchased from Thermo Fisher at concentration of 1.0 nmol μL^–1^. (Fisher Scientific, Toronto, ON, Canada). Chromatographic behavior and fragmentation spectra of standards were also confirmed by LC-MS/MS analysis. The reference standards were spiked into samples at a final concentration of 125 fmole μL^–1^ to quantify the candidate proteins and isomers. Integration of MRM transitions (peak areas) was performed using MultiQuant (Vison.2.1.1. AB Sciex, Toronto, ON, Canada) and exported for further statistical analysis (25 and 26).

### Statistical Analysis

The analysis was completed using a mixed model procedure (ANOVA) in GenStat (16th edition, VSN International, Hemel Hempstead, United Kingdom). The random effects used were harvest with cultivars and maturity used as fixed effects within a randomized block design. All data was tested for normality resulting in a log transformation being used and a significance cut off of ≤0.05. The cluster software^3^ was applied to the proteomic data. Proteins were clustered according to their expression profile across cultivars and ripeness stages (25 and 26).

## Results

### Identification of Proteins and Characterization of Peptide Transitions Involved in Antioxidant Defense System in Strawberry Fruit

#### Identification of Protein

Protein identification in strawberry fruit was conducted on a data set collected previously from OGE with 24 fractions (Yang et al., 2014). Based on the identifications and characterization, 62 proteins and their corresponding peptide transitions in association with antioxidant enzymes were selected for the MRM study. Targeted protein and peptide information is provided in Supplementary Table S1.

#### Evaluation of Peptide Transitions

Only the peptides that were identified and associated with antioxidant enzymes/related proteins were further selected and characterized for the MRM study. MRM transitions were detected and optimized (Supplementary Table S2).

In total, we identified 105 strawberry proteins from 24 fractions of OFFGEL as our initial targets for the MRM study. Only peptides with at least four transitions and without any modification were selected and optimized. Ultimately, 73 peptides transitions with optimized CE were established and used for proteins and isoforms related to strawberry antioxidant enzymes. Information on the MRM transitions of peptides, Q_1_/Q_3_ transitions and proteins are provided in Supplementary Table S2. Based on the detection of peptide transitions from all 24 OGE fractions, Some fractions were combined into three groups of fractions (1–5, 9–15, and 19–24).

### Quantitative Changes in Proteins Involved in Antioxidant Defense System in Strawberry in Association With Fruit Maturity and Cultivar

**FIGURE 1:**
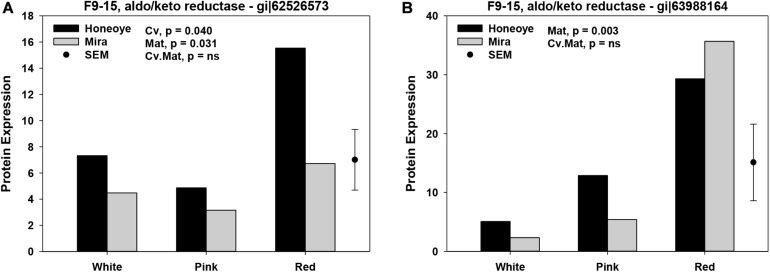
Significantly increased proteins and isomers in association with antioxidant enzymes of strawberry fruit at three ripening stages. Protein expression was calculated based on the intensity of MRM transitions of protein and weighed average from corresponding peptides. **(A)** Significant increased abundance of protein and isomers in fractions 1–5; **(B)** Significant increased abundance of protein and isomers in fractions 9–15 and 19–24. The random effects used were harvest with cultivars (Cv) and maturity (Mat.) as fixed effects. SEM: standard error of mean.

Significant changes in abundance of some protein and isomers in association with redox and antioxidant enzymes were found. ANOVA analysis revealed that maturities resulted in significant changes (*p* < 0.05) of 12 proteins and isomers from three groups of combined fractions. In fraction group 1–5, protein abundance of AKR and isomers (gi| 53988164 and gi| 255542314), superoxide dismutase (SOD) (gi| 470102209). two APXs (gi| 5257500 and gi| 189163449), as well as glutathione reductase (GR) (gi| 470121124) increased significantly as fruit ripened from white to red stage (Figure 1A). In fraction group 9–15, two AKRs (gi| 62526573 and gi| 63988164) and glutathione transferase (GT) (gi| 476490875) significantly increased. In addition, another AKR isomers (gi| 63988164) was found to increase in fraction group 19–24 (Figure 1B).

**FIGURE 2:**
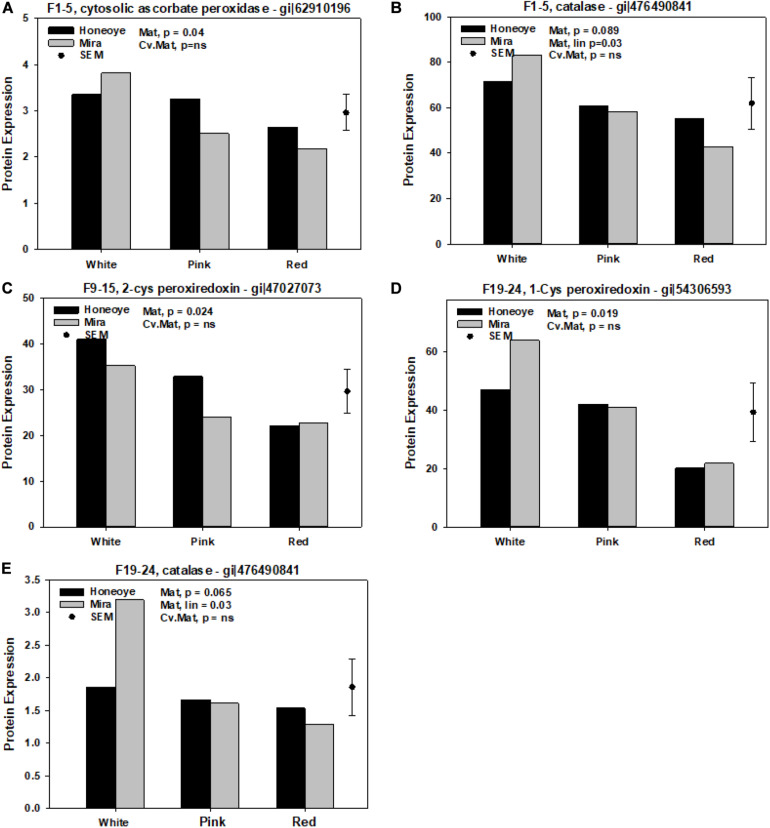
Significantly decreased proteins and isomers in association with antioxidant enzymes of strawberry fruit at different ripening stages. Protein expression was calculated based on the intensity of MRM transitions of protein and weighed average from corresponding peptides. The random effects were harvest with cultivars (Cv.) and maturity (Mat.) used as fixed effects. **(A,B)** Significantly decreased abundance of protein and isomers in fractions 1–5. **(C)** Significantly decreased abundance of protein and isomers in fractions 9–15. **(D,E)** 19–24. SEM: standard error of mean.

On the other hand, significantly reduced protein abundance was determined from all fractions (Figure 2). Cytosolic ascorbate peroxidise (CAP) (gi| 62910196) decreased significantly in fraction 1–5. Meanwhile, 2-Cys peroxiredoxin (gi| 47027073) and 1-Cys peroxiredoxin (gi| 54306593) decreased significantly in fraction 1–5 and 19–24, respectively in red as compared to white stage (Figure 2). In addition, catalase (CAT) (gi| 476490841) decreased in ripe fruit (at red stage) in fraction 1–5 and 19–24, although the decrease of CAT (gi| 476490841) was only significantly in “Mira.”

Differences in some proteins and isomers were found between “Mira” and “Honeoye.” Abundance of GR (gi| 470121124), AKR (gi| 53988164), monodehydroascorbate reductase (gi| 4760483), peroxiredoxin-2B (gi| 470128097) as well as thioredoxin h (gi| 71534922) was higher in “Honeoye” than in “Mira.” However, changes in interaction between cultivar and maturity was not significant.

### Hierarchical Cluster Analysis of Differentially Changed Proteins and Isomers

**FIGURE 3:**
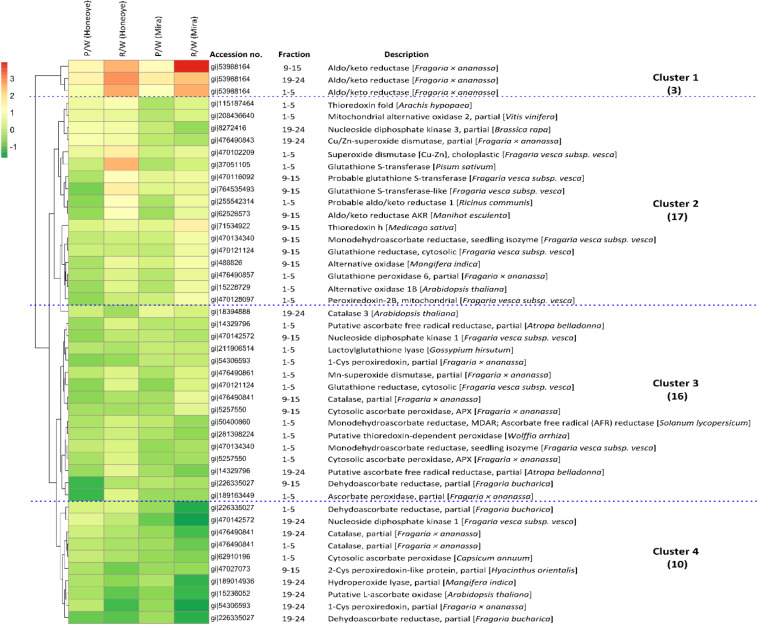
Hierarchical cluster analysis of 46 proteins and isoforms involved in antioxidant and redox enzymes in strawberry fruit (“Honeoye” and “Mira”) at three different maturities. The ratio between pink/white and red/white was calculated as log2 fold changes. Four major clusters of protein changes were demonstrated: Cluster 1: increased proteins and isomers in both “Honeoye” and “Mira” in both pink and red fruit; Cluster 2: differently changed proteins and isomers between “Honeoye” and “Mira.” Cluster 3: decreased protein and isomers differently in “Honeoye” and “Mira” in pink and red fruit; Cluster 4: decrease protein and isomers in both “Honeoye” and “Mira” in both pink and red fruit. Increasing intensities of red or green color represents differentially increase and decrease abundance of protein and isomers as compared with the control at white stage, respectively.

Employing cluster analysis, the dynamics and correlations of the protein abundance profiles from this study are shown (Figure 3). In total, there are four clusters that can be demonstrated. The first cluster constitutes only one protein, an AKR and its isoforms that increased significantly in abundance from white to red stage and showed high abundance in red fruit. The second cluster contains 17 proteins and isomers with increasing trends slightly during ripening but showed some difference between the “Mira” and “Honeoye” cultivars. Among them, key proteins such as SOD [Cu-Zn] and glutathione synthase (GST) up-regulated in pink and red fruit as compared to white fruit, especially in “Honeoye.” The third cluster includes 16 proteins and isoforms with decreased abundance in both cultivars and with notably decreased abundance only in red ripe fruit. The fourth cluster contains 10 proteins and isomers that decreased significantly during ripening. This cluster includes CAT, APX, 1-cys peroxiredoxin (1-Cys PRX), 2-cys peroxiredoxin (2-Cys PRX), nucleoside diphosphate kinase 1, ascorbate oxidase and dehydroascorbate reductase, which decreased in both cultivars. Analysis of these proteins demonstrated dynamic changes and correlation of antioxidant enzymes to different fruit ripening stages.

## Discussion

Eating quality plays an important role in fruit and vegetable and is comprised of major characteristics including appearance, texture, color, flavor and nutritional components. Despite for the development of technologies to maintain and improve the quality, fundamental research is necessary to reveal regulating mechanism of quality of fruits and vegetables at gene, protein and metabolite levels. Strawberry fruit undergo many significant physiological changes during maturation that drive color formation (through anthocyanin biosynthesis), softening, sugar accumulation, acid loss, and flavor volatile production. Despite the various genes that have been identified related to fruit ripening in strawberries, many of the biosynthetic pathways associated with fruit quality are not fully understood. Although strawberry fruit are considered to be non-climacteric, it showed a different pattern of hormones changes as other non-climacteric fruit such as grape during ripening. Therefore, it is proposed that there may be no consistent hormone changes for non-climacteric fruit due to the variation of fruit structure and phylogenetic relationships (Symons et al., 2012). Strawberry fruit behave differently from climacteric fruit in terms of ACO gene expression and response to auxin treatment (Symons et al., 2012). Therefore, fundamental metabolomic investigations into the role of factors other than ethylene production or reception are needed to increase our understanding of strawberry fruit ripening (Wang et al., 2017).

It has been proposed that fruit ripening is associated with metabolites of antioxidants that respond to or process ROS (Jimenez et al., 2002). Molecular and genomic investigations have also been conducted on redox and antioxidant enzymes including CAT, APX, GST, GR, and peroxidase that respond to ROS and play a role in stress in strawberry fruit (Aharoni et al., 2002). Reduced accumulation of ROS levels was observed in tomato fruit of the ripening mutants *rin*, *nor*, and *Nr* compared to the wild type fruit, as well as down regulation of CATs and CuZnSOD. Gene expression in association with maintaining cellular redox state was found which provided additional evidence for the involvement of ROS in tomato ripening and senescence (Kumar et al., 2016). Most prior research has focused on the measurement of enzyme activity and gene expression. A few proteomic studies employing a gel-based approach and labeling analysis reported some quantitative changes in proteins and isomers in relation to redox and antioxidant system in strawberry fruit (17 and 18). To our knowledge, there has been no targeted quantitative analysis at the protein or isomer level to comprehensively investigate quantitative changes in all enzymes and isomers due to technical limitations.

Multiple reaction monitoring technologies offers significant advantages in good sensitivity and accurate quantification of proteins/peptides in a biological sample (Rödiger and Baginsky, 2018). Based on the results from LC/MS proteomic analysis, MRM assays can be characterized and established for a set of peptides that can be monitored and quantify protein changes in certain metabolic pathways. Protein abundance changed in association with increased volatile production, color formation (biosynthesis of anthocyanins) and other fruit maturity indicators as previously reported (Song et al., 2015a, b). Recently, MRM techniques have been employed to quantify ethylene receptors (CTR and EIN2) during tomato fruit ripening (Mata et al., 2018).

Fruit at harvested at different maturities showed significant differences in quality indices (Li et al., 2013). Significant increase of soluble solids content (SSC) increased to 7.6 and 8.6% in “Honeoye” and “Mira,” respectively was found. Meanwhile, decrease of titratable acidity (TA) by 50% in red fruit was reported. Total anthocyanin content, representing cyanidin-3-glucoside or pelargonidin-3-glucoside, increase 200–300% in red as compared to white fruit (Li et al., 2013).

In the present study, we investigated the quantitative changes in redox and antioxidant system proteins during strawberry fruit ripening. We found that AKR and its isomers increased significantly during fruit ripening, with similar trends found in SOD, APXs, GR, and GSTs as fruit ripened from white to red. In contrast, we observed a significant decrease in abundance of cytosolic APX, 1-Cys and 2-Cys PRX and CAT.

The AKRs belong to the superfamily of NADP (H) dependent oxidoreductases, comprised of a large number of primarily monomeric proteins. Some major and important functional roles of AKRs in plants has been summarized as reactive aldehyde detoxification, biosynthesis of osmolytes, secondary metabolism and membrane transport (Sengupta et al., 2015). In addition, AKRs have functions as biotic and abiotic stress defense and production of commercially important secondary metabolites in plants (Suekawa et al., 2016; Vemanna et al., 2017). In strawberry, an AKR isozyme was classified as AKR4B and was reported to be involved in an increase of ascorbic acid during fruit ripening and linked with remobolizing hydrolytic pectin compounds (Agius et al., 2002). In tomato fruit, gene expression of AKR 4B was detected in senescent leaves and ripening fruit. It did not correlate with L-ascorbate content, but was induced by hydrogen peroxide (H_2_O_2_), NaCl, and plant hormones such as salicylic acid and jasmonic acid, suggesting AKR is involved in stress response. In addition, it was determined that the promoter region of SIAKR4B contains *cis*-elements for abiotic stress induced response (Sengupta et al., 2015). At this point, it is very difficult to explain the exact role of AKR in strawberry fruit ripening, however, the significant increase of AKRs in association with ripening is clearly evident in this study. This reflects the complex nature of fruit development and the participation of AKRs in this process, which may involve stress response, hormone interaction and/or substrate metabolism. In addition, key enzymes such as SOD [Cu-Zn] and GST were also found to up-regulate in pink and red fruit compared to white fruit, especially in the cultivar “Honeoye.” In general, plant stress is characterized by an increase of reactive oxygen species (ROS) that are harmful to plants, requiring an efficient ROS scavenging system to maintain redox system balance. Antioxidant enzymes such as SOD, APX, and GR are among the dominant H_2_O_2_ scavengers in chloroplasts and mitochondria (Foyer et al., 2020). Gene expression during grape berry ripening showed an increase of a Cu/ZnSOD and a MnSOD among the 12 investigated SODs (Guo et al., 2020). SOD has been reported to increase in apple fruit during ripening (Zheng et al., 2007). While in another study on apples, SOD activity in both pulp and peel increased and later decreased, which coincided with the change of respiration rate and H_2_O_2_ content (Lv et al., 2020), indicating that ethylene may play a role in the regulation of gene expression of SOD in apples.

In addition, APX was reported to increase during ripening of strawberry fruit (Pandey and Mann, 2004). The increase of APX was confirmed in this study. Two GSTs were also found to increase during fruit ripening (Figure 1). GSTs belong to a super family of proteins encoded by a large gene family (Mhamdi et al., 2010). GSTs have been reported to respond to a wide range of biotic stresses and function to detoxify toxic substances, respond to stress and pathogen attack, and attenuate oxidative stress (Foyer et al., 2020). There is also a tight link between GSTs and plant defense hormones. GSTs response to auxins and played a role in plant secondary metabolism of such as anthocyanins and cinnamic acid (Marrs, 1996). GST was repressed by auxin and identified as one of the ripening regulated genes during strawberry fruit ripening (Aharoni et al., 2002).

Enzymes such as CAT and peroxidase also have a role in removing H_2_O_2_ and free radicals. A significant decrease of CAT and isomers 1-Cys and 2-Cys PRX was found in this study. CAT is a ubiquitous oxidoreductase in plant peroxisomes that decomposes H_2_O_2_ to water and molecular oxygen and removes toxic peroxides during photorespiration (Foyer et al., 2020). Environmental stress can reduce the activity of CATs under conditions such as chilling, drought and hypoxia (Mhamdi et al., 2010). Decrease of *CAT2* gene expression in *Arabidopsis* plants after flowering was shown, suggesting an integral part in H_2_O_2_ triggered leaf senescence (Zimmermann et al., 2006). Reduction in CAT activity under stress conditions has been attributed to the inactivation of enzymes due to ROS (Lv et al., 2020). Measuring the activities of CAT, SOD and peroxidase in “Chandler” strawberry fruit during development and fruit ripening reported that activity of CAT increased, while SOD and peroxidase decreased from white to red ripe fruit (López et al., 2010). A decline in abundance of CAT under the present study may suggest a reduced capacity in removal of H_2_O_2_ and toxic peroxides which may resulted in an increase in the free radical initiation of lipid peroxidation with fruit ripening. In the CAT deficient lines of *Arabidopsis* plants, GSTs were induced (Lv et al., 2020). This relationship between CAT and GSTs is confirmed in this study and may reflect a simple association with the loss of chloroplast function as the fruit ripen from white to red. However, more biochemical evidence is required to reveal the dynamic changes in CAT and its interaction with other antioxidant enzymes.

Peroxiredoxins (Cys-PRXs) are important plant specific peroxide detoxifying enzymes whose roles include acting as peroxidases, chaperones, transmitters of redox signal and degradation of H_2_O_2_. Of the two Cys-PRX identified, 1-Cys-PRX is found in the cytosol, while 2-Cys-PRX is found in the chloroplast (Dietz et al., 2006). The 2-Cys PRX seems to serve an unique function in photosynthesis and many metabolic pathways in chloroplast (Koenig et al., 2002). Research strongly supports that PRX plays an important role to the detoxification of Mehler-derived H_2_O_2_ in the chloroplast through mediated peroxide reduction and as an alternative to APX. Its role in ROS detoxification network in chloroplast should also be considered (Dietz et al., 2006). Research indicates that Cys PRX related post-translational modifications (PTMs) are also essential regulators of plant stress signaling and ROS (Foyer et al., 2020). In the present study, both Cys-PRXs isomers decreased significantly during strawberry fruit ripening, which agrees with the theory that Cys-PRX content is negatively related to fruit ripening (Li et al., 2013). The decrease in Cys-PRXs may be also linked with the decrease of CAT, simply due to the metabolic activities shifting away from photosynthetic activity as the fruit turns from white to pink to red. Significantly higher amounts of L-ascorbic acid, with higher transcriptional activation of the L-galactose pathway, were identified in green (immature) achenes compared to red fruit (Aragüez et al., 2013). However, considering the possibility of signal transduction of ROS and PRX (Nikkanen et al., 2016), it is also possible that the significant changes in redox and antioxidant activities not only maintain the ROS homeostasis of the fruit cell, but also contribute to signal transduction involving fruit ripening. Further research is required to investigate the complex regulatory network of antioxidant enzymes involved in non-climacteric fruit with low ethylene production during fruit ripening and senescence.

## Conclusion and Future Perspectives

Significant research has been conducted on antioxidant capacity and phenolic compounds in strawberry during ripening, but little is known about the changes in redox and antioxidant enzymes. Despite intensive efforts using molecular biology tools in the past, it has been a challenge to fully understand the molecular mechanisms controlling fruit ripening. To explore the possible biological effects of the antioxidant enzyme system in strawberry fruit, a targeted quantitative proteomics approach employing LC-MS analysis and MRM was conducted. This study reveals significant quantitative changes of antioxidant enzymes (redox) in ripening strawberry fruit. This targeted approach, which used MRM on multi-targeted proteins, allowed us to investigate the multiple proteins and isomers simultaneously and provides direct quantitative evidence of the dynamic changes in these enzymes during fruit ripening. The results support previous reports that had lacked quantitative proteomics, as well as provide novel insight through quantitative proteomic evidence on the control of the antioxidant (redox) pathway that occur during strawberry fruit ripening.

## Data Availability Statement

The data presented in the study in the PASSEL (www.peptideatlas.org/passel/) repository accession number PASS01641.

## Author Contributions

JS conceived, designed, and supervised the study. LC conducted the experiment and collected the data. LC, MV-T, SF, and HL conducted the data analysis and interpretation. JS wrote the manuscript with input from CF and ZZ. All authors read and approved the final manuscript.

## Conflict of Interest

The authors declare that the research was conducted in the absence of any commercial or financial relationships that could be construed as a potential conflict of interest.

## Footnotes

1 http://www.ncbi.nlm.nih.gov/

2 http://proteome.gs.washington.edu/software/skyline

3 http://biit.cs.ut.ee/clustvis/

## Abbreviations

AKR: aldo/keto reductase
APX: L-ascorbate peroxidase
CAT: catalase
GT: glutathione transferase
GST: glutathione synthase
GR: glutathione reductase
H_2_O_2_: hydrogen peroxide
MRM: multiple reaction monitoring
PRXs: 1-Cys peroxiredoxin and 2-Cys peroxiredoxin
ROS: reactive oxygen species
SOD: superoxide dismutase.
